# Descriptive study on ocular survival, visual outcome and prognostic factors in open globe injuries

**DOI:** 10.4103/0301-4738.64116

**Published:** 2010

**Authors:** Lavanya G Rao, Anju Ninan, Krishna A Rao

**Affiliations:** OEU Institute of Ophthalmology, Kasturba Medical College, Manipal, India

**Keywords:** Outcome, penetrating eye injury, prognostic factors

## Abstract

A prospective observational study was done to assess ocular survival, visual outcome and prognostic factors of open globe injury. Eighty eyes of penetrating trauma between 2004 and 2006 were categorized according to the ocular trauma classification system. Primary repair was done and outcomes were assessed at one, three and six months. The final vision was categorized as per World Health Organization classification of visual impairment. Factors at presentation were evaluated for prognostic value towards visual outcome. Sixty-nine eyes with minimum one month follow-up were included for analysis. Statistical analysis was done using Univariate and Multivariate analysis. We found Grade IV visual acuity (<5/200) at presentation (64%) as the most important factor contributing to poor visual outcome. Statistically insignificant factors were time since injury, cataract, and presence of intraocular foreign body. Ocular survival was 97%. We concluded that initial visual acuity, hyphema, zone and length of injury, retinal detachment and vitreous hemorrhage are statistically significant factors affecting outcome in open globe injuries.

Knowledge of the prognostic factors help the physician in making decisions regarding patient management and rehabilitation.[1] Factors reported to correlate with visual outcome were:[1] type, location, extent of injury, initial visual acuity, afferent pupillary defect, lenticular involvement, vitreous hemorrhage, intraocular foreign body. In this study we have evaluated visual outcome, ocular survival, prognostic factors and complications of open globe injuries.

## Materials and Methods

This was a prospective observational study of 80 cases of penetrating trauma which presented to our hospital between November 2004 and August 2006. Relevant history included patient details, mechanism, and time since injury. Examination was done to detect initial visual acuity (Snellen's acuity), length of wound, zone of injury, presence of iris prolapse, afferent pupillary defect, cataract, hyphema, retinal detachment, vitreous hemorrhage, intraocular foreign body. These factors were categorized according to ocular trauma classification system.[2] Primary repair was done under general anesthesia. Patients were reviewed at one, three and six months. Assessment included best corrected visual acuity, wound status, intraocular pressure (IOP), fundus examination and B-scan ultrasonography. Final visual acuity was graded according to World Health Organization (WHO) visual impairment categories:

≥ 20/70, (good visual outcome), <20/70 – 20/200, <20/200 – 20/400, <20/400 (low visual outcome)

Eleven eyes were lost to follow-up and 69 eyes were included for statistical analysis. Eight eyes had only one month follow-up. Univariate analysis was done using Chi Square test, multivariate analysis was done to find prognostic significance. *P* value <0.05 was considered significant.

## Results

**Figure 1:**
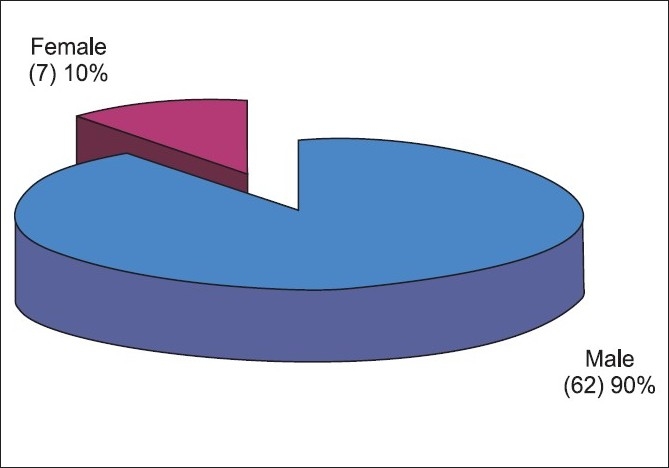
Sex distribution of the study population

**Figure 2:**
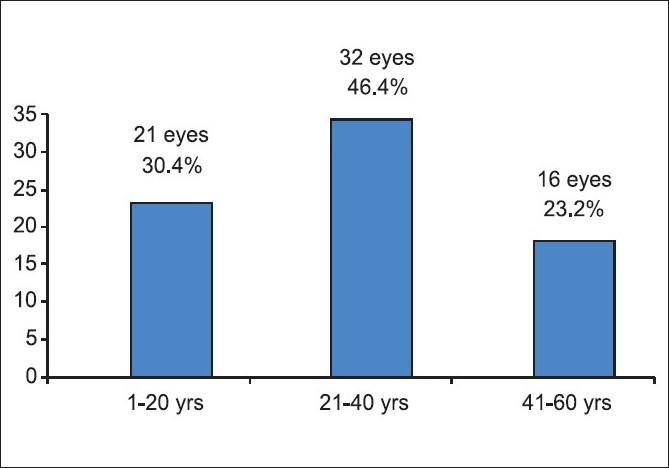
Age distribution of the study population

**Figure 3:**
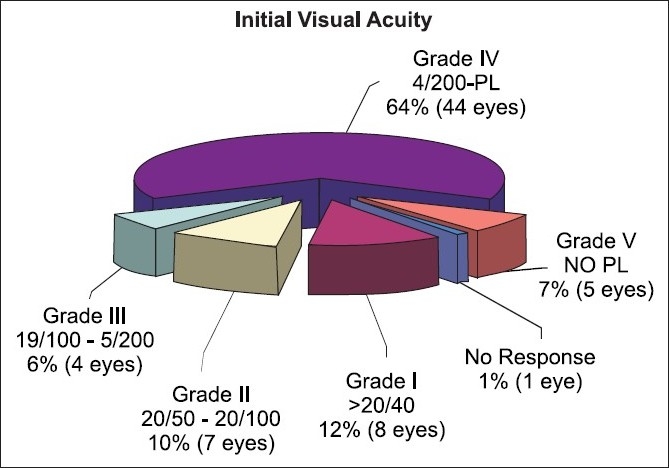
Grade of injury

**Figure 4:**
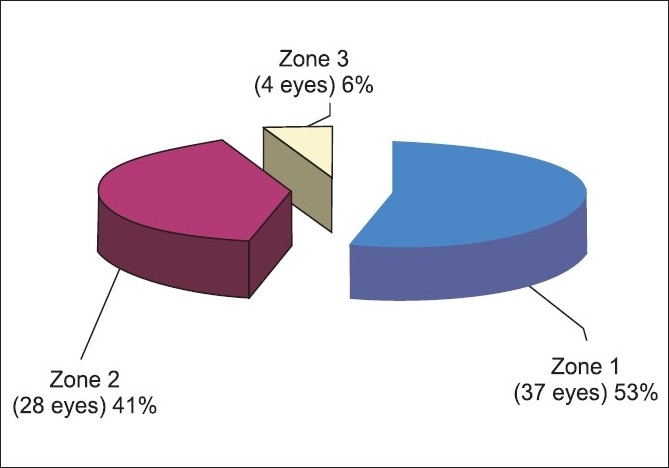
Zone of injury

**Figure 5:**
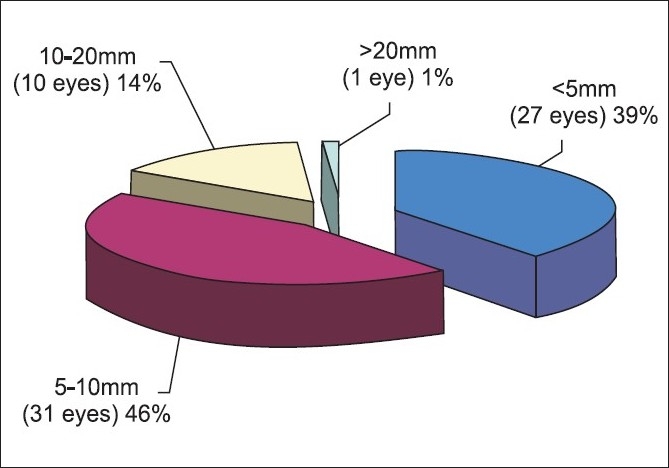
Length of wound

The age, sex distribution, grade, zone of injury and length of wound have been shown in Figs. 1‐5. One four-year-old child, was not cooperative for assessment and was placed under no response [Fig. 3]. Afferent pupillary defect in one eye with no perception of light was eviscerated .Foreign bodies were found in 11 eyes (16%), four corneal, one in iris, six in vitreous, foreign bodies were removed during primary repair, one eye was eviscerated.

Endophthalmitis was found in eight eyes (12%), out of which seven were at presentation and in one eye after a month. Six eyes received intravitreal vancomycin and ceftazidime. Two eyes, which progressed to panophthalmitis were eviscerated. Four of 6 eyes had final visual acuity < 20/400. One eye had a good outcome of 20/30.

Hyphema was noted in 29 eyes (42%), and was drained during primary repair. Lens opacities were found in 28 eyes (41%). Fourteen had cataract extraction. The remaining 14 had useful vision in spite of lens opacities. Iris prolapse was found in 40 eyes (58%) (abscission - 30 eyes, iris released - 10 eyes). Retinal detachment was found in four eyes (6%). Vitreous hemorrhage was found in nine eyes (13%).

Visual outcomes at one, three and six months have been shown in Table 1.

**Table 1:** Visual outcome at 1, 3 and 6 months

Vision at 1 months			Vision at 3 months			Vision at 6 months		
Vision	Eyes	%	Vision	Eyes	%	Vision	Eyes	%
Eviscerated	2	2.9	Eviscerated	2	2.9	Eviscerated	2	2.9
			No follow-up	8	11.6	No follow-up	9	13.0
≥ 20/70	21	30.4	≥ 20/70	24	34.8	≥ 20/70	24	34.8
<20/70 – 20/200	9	13.1	<20/70 – 20/200	9	13.1	<20/70 – 20/200	9	13.0
<20/200 – 20/400	4	5.8	<20/200 – 20/400	1	1.4	<20/200 – 20/400	1	1.4
<20/400	33	47.8	<20/400	25	36.2	<20/400	24	34.8
Total	69	100	Total	69	100	Total	69	100

Ocular survival at the end of the study period was 97% (67 eyes). Two eyes were eviscerated. Six eyes had phthisis, nine eyes had raised IOP, three underwent combined surgery and the rest were put on medical management.

## Discussion

Average age was 30 years (4-58 years). Seventy per cent patients were below 40 years of age. Statistical analysis at one month showed age as a significant factor (*P* <0.001) for predicting visual outcome. The age distribution is similar to other studies. Usha Vasu found 81% patients below 45 years of age.[3] Sternberg *et al*, found age as a significant factor affecting visual outcome.[4]

In our study 90% patients were males and 10% females. Gender was found to be a statistically significant factor (*P*<0.001). William *et al*,[5] had 98% male patients, Vasu *et al*, found 95% were males.[3] This could be attributed to increased outdoor activities of males.

The most important factor of prognostic significance in penetrating injury is the initial visual acuity. Sternberg *et al*, found initial visual acuity >20/800 as the most important factor for favorable prognosis.[4] Williams *et al*,[5] Barr *et al*,[6] Esmaeli *et al*,[7] concluded that initial visual acuity is an important prognostic indicator of visual outcome. Using univariate analysis, initial visual acuity had statistically significant influence on the outcome at one, three and six months. Multiple logistic regression proved this factor to be statistically significant (*P* <0.001) at one month.

Charles Barr found hyphema to be a predictor of visual outcome.[6] We found hyphema significant (*P*<0.001) at one month. Of these, 76% at one month, 55% at three and six months had poor outcome (<20/400).

The second important prognostic factor is length of the wound.[5‐7] By multivariate analysis it was a prognostic factor at one and six months (*P*<0.001).

Many reports mention lens damage as a predictor of outcome.[1,6,8] We found statistically no significant predictive value for lens damage by univariate, multivariate analysis.

Hutton *et al*[9] and Sternberg *et al*,[4]found that corneal wounds have better result than the others. By multivariate analysis the zone of injury was a significant predictor at one, three and six months (*P* = 0.004 at one month, *P* < 0.001 at three and six months) .Statistical analysis did not show intraocular foreign body as a predictive factor in our study, but 71% patients with foreign body had poor visual outcome (<20/400).

Endophthalmitis has been mentioned as a prognostic indicator by William *et al*[5]and Brinton *et al*.[10] The association in our study was statistically significant only at one month (*P* = 0.009).

Retinal detachment was found as a significant prognostic factor by Hutton *et al*[9] and Thompson *et al*.[8] We found that 100% patients with retinal detachment had poor visual outcome <20/400, showing it to be a significant prognostic factor (*P* <0.001) at three and six months.

Vitreous hemorrhage was found as a prognostic factor.[1,6] In our study significant association was found at one and six months (*P* <0.001).

In our study 64% eyes came with a poor initial vision of <5/200. Young males (15-40 years) were most commonly affected, average age being 30 years. At six months, 35% had a good visual outcome of >20/70 and an equal number had a poor visual outcome of <20/400. In our study significant predictors of outcome were initial visual acuity, hyphema, age, length of wound and zone of injury, retinal detachment and vitreous hemorrhage. Time since injury, cataract and intraocular foreign body were found to be insignificant for assessing the prognosis. The ocular survival rate was 97%.
